# Blood vessel density correlates with the effects of targeted intra-arterial carboplatin infusion with concurrent radiotherapy for squamous cell carcinomas of the oral cavity and oropharynx

**DOI:** 10.1038/sj.bjc.6603138

**Published:** 2006-05-02

**Authors:** S Takagi, R Inenaga, R Oya, S Nakamura, K Ikemura

**Affiliations:** 1 Department of Oral and Maxillofacial Surgery, School of Medicine, University of Occupational and Environmental Health, Kitakyushu, Japan

**Keywords:** blood vessel density, targeted intra-arterial chemotherapy, carboplatin, radiation therapy, oral cavity cancers

## Abstract

Our aim was first to evaluate the association between blood vessel density (BVD) and free platinum concentration in experimentally induced tumours in rabbits. We also investigated the association between tumour BVD and the clinical response of patients who had undergone targeted carboplatin intra-arterial (i.a.) chemoradiotherapy. VX2 carcinoma cells were transplanted into 46 inbred female Japanese white rabbits. In the i.a. group, carboplatin was infused into the lingual artery, and in the intravenous (i.v.) group, carboplatin was infused through the auricular vein. In the clinical study, we evaluated 19 patients with squamous cell carcinomas of the oral cavity and oropharynx, who had undergone targeted carboplatin i.a. chemoradiotherapy and had been administered i.a. tegafur/uracil chemotherapy before surgery. We quantified angiogenesis in both studies. Increased BVD was associated with a higher free platinum concentration in the tumour region in the i.a. group of rabbits. In the clinical study, using multivariate logistic regression analysis, only the BVD was related independently to the treatment effect. Therefore, BVD is a valid predictor of the effects of i.a. targeted carboplatin chemotherapy and concurrent radiotherapy for treating human oral and oropharyngeal squamous cell carcinomas.

The anticancer effect of superselective chemotherapy is believed to be reinforced when highly concentrated drugs are administered to tumours via supplying arteries (Kar *et al*, 1986; Moses *et al*, 1993). However, little evidence has been reported from experimental trials in the oral region. We also hypothesise that the concentration of anticancer drug in a tumour may be associated with the blood vessel density (BVD) in the tumour. Consequently, BVD may influence therapeutic effects, in that different treatment effects may be caused partly by differences in the degree of vascularity of a tumour. Much experimental evidence has shown that tumour growth and metastasis depend on local angiogenesis (Folkman and Klagsbrun, 1987; Horak *et al*, 1992; Macchiarini *et al*, 1992) but no study has elucidated the relationship between the effects of chemotherapy and tumour angiogenesis. This study was designed to evaluate the relationship between BVD and free platinum concentrations during intra-arterial (i.a.) chemotherapy. Using transplanted VX2 tongue tumour cells in Japanese white rabbits, and a series of 19 human patients, we evaluated the association between BVD and the effects of concurrent targeted i.a. carboplatin chemotherapy and radiotherapy before surgery in squamous cell carcinomas of the oral cavity and oropharynx.

## MATERIALS AND METHODS

### Experimental study

Inbred female Japanese white rabbits weighing 3–4 kg were used throughout the experiment. The animals were anaesthetised via the auricular vein using pentobarbital sodium (1 mg kg^−1^), and then VX2 carcinoma cells were implanted as 0.2 ml injections of cell suspension into the tongue, using a 22-gauge needle. By 3 weeks after implantation, the tumours had grown to 2.0 cm^2^, which enabled us to evaluate the free platinum concentration.

**Figure 1:**
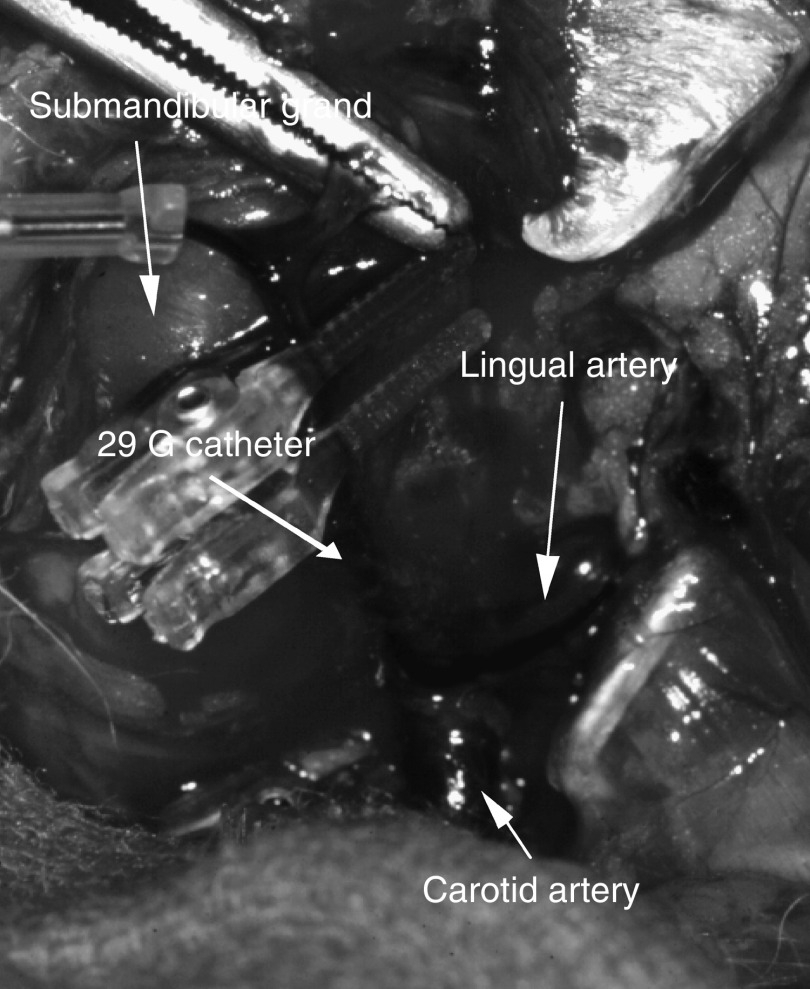
Intra-arterial infusion was accomplished by incising the midline of the neck. After the left carotid and facial arteries were isolated, a 29-Gauge catheter was introduced into the tongue artery by way of the submandibular branch.

**Figure 2:**
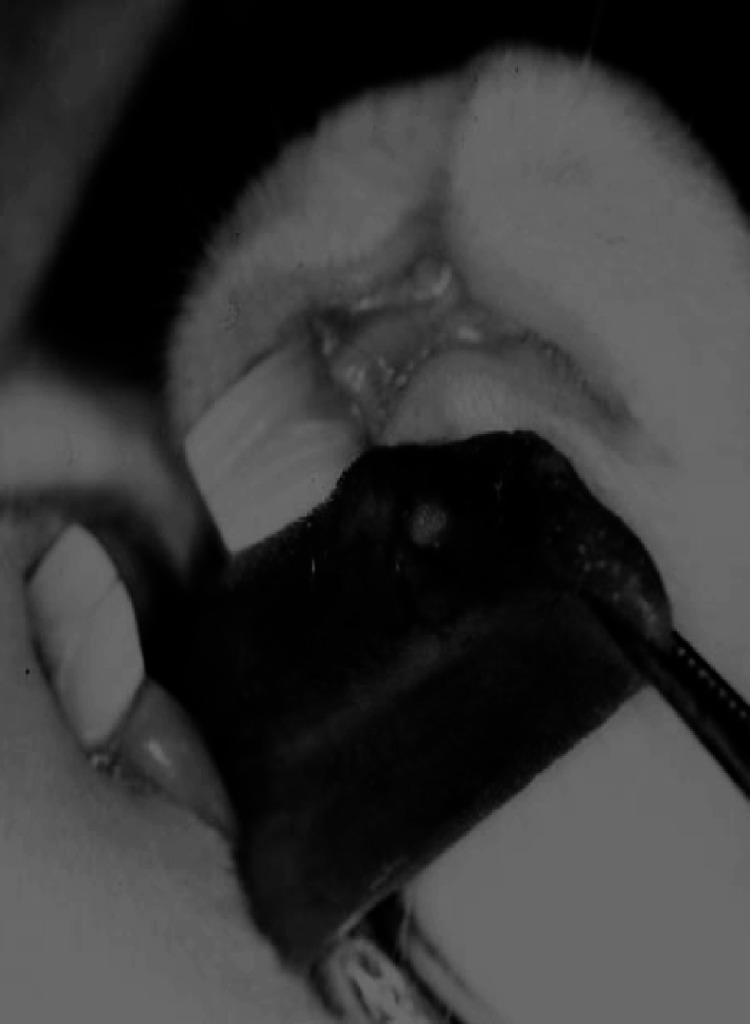
The injection of indigocarmine via the catheter indicated whether the infused carboplatin appropriately access the tongue at the cancerous site.

**Figure 3:**
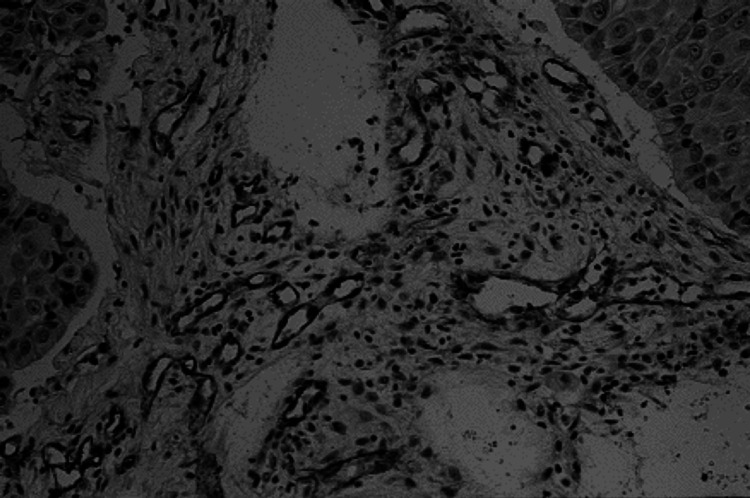
Microvessel staining. Blood microvessels were stained with CD31 using a standard immunoperoxiderse-staining technique. All sections were routinely processed, cut into 4 *μ*m thick sections and then were stained using the LSAB (labelled streptavidin–biotin) method.

For i.a. carboplatin infusion, a 29-gauge catheter was introduced into the lingual artery on the VX2-transplanted side in 26 rabbits kept under general anaesthesia. Access to this artery was gained via the arterial branch of the submandibular gland (Figure 1). The catheter was connected to tubing and the drug was injected with a constant infusion pump. Indigo carmine was injected via the catheter to confirm whether the infused carboplatin would be appropriately distributed in the tongue (Figure 2). Carboplatin was then infused at a dose of 6 mg kg^−1^ of body weight over about 20 s. Heparin (5000 U l^−1^) and methylprednisolone (2 mg animal^−1^) were added to prevent thromboses and spasms at the catheterisation site. For intravenous (i.v.) carboplatin delivery, a similar catheter was positioned in the auricular vein on the VX2-transplanted side and the same dose of carboplatin was then infused. To measure the concentrations of blood vessels, we excised tongue tissue specimens in the tumour and normal regions immediately after the injection of carboplatin. The blood vessels were stained with factor-VIII-related antigen using a standard immunoperoxidase technique (Figure 3). The most vascular areas of the tumour were viewed at low magnification, and the vessels were counted in four fields at × 200 (corresponding to an area of 0.16 mm^2^). The highest vascularity count of four fields was used as the final score.

To measure free platinum concentrations in tissues, we excised tongue tissue specimens immediately after and at 24 h after the injection of carboplatin. All rabbits were then euthanised and the kidneys and livers were removed. The free platinum concentration was measured using an atomic absorption spectrometer (Sumitomo Material Bioscience Corp, Tokyo, Japan).

This experimental study conformed to the ethical guidelines for animal experimentation of the University of Occupational and Environmental Health, Kitakyushu, Japan.

### Clinical study

Nineteen patients with squamous cell carcinomas of the oral cavity and oropharynx, who had undergone targeted i.a. carboplatin infusions with concurrent radiotherapy and the oral administration of tegafur/uracil (UFT®) before surgery at the University Hospital of Occupational and Environmental Health, Japan, were examined between April 1995 and February 2002 (Table 1). All patients included in this study had to have previously untreated, histologically proven squamous cell carcinomas of type T3, T4 or T2 (a strong tendency to infiltration and a diameter of greater than 3 cm), arising from the oral cavity and oropharynx, with any N status, but without distant metastases. Patients with a performance status of 0–1 according to the World Health Organization scale were eligible. All patients also had to have a serum creatinine concentration of 2.0 mg dl^−1^ or less, a white-cell count of at least 4000 *μ*l^−1^, a platelet count of at least 100 000 *μ*l^−1^, and a haemoglobin (Hb) concentration of at least 10.0 g dl^−1^. In addition, the AST and ALT values needed to be less than 50 IU l^−1^ with bilirubin values less than 2.0 mg dl^−1^. All patients provided informed consent for participation in this treatment. The median age of the patients was 67 years (range, 43–78; mean 63.1); six were women and 13 were men. These patients were staged according to the UICC–TNM staging system (Hermanek and Sobin, 1987), based on the pattern of invasion described by Anneroth *et al* (1987). This consisted of four categories: *Grade 1,* tumours showed invasive, well-delineated borders; *Grade 2*, infiltrating, solid cords, bands and strands; *Grade 3*, small groups or cords of infiltrating cells (*n*>15); *Grade 4*, marked and widespread cellular dissociation in a small group of cells (*n*<15) and/or in single cells. We recorded the lowest Hb levels observed during chemoradiation. Haemoglobin concentrations were classed as low (⩽11.5 g dl^−1^) or high (>11.5 g dl^−1^). All patients were followed up for at least 2 years.

All patients in the series were administered oral tegafur/uracil (400–600 mg day^−1^), from the time histological diagnosis was determined until the completion of their courses of radiation therapy and chemotherapy. For the carboplatin infusions, a microcatheter was introduced angiographically by a radiologist to the artery supplying the tumour. Because occasionally the tumour stain could not be observed with digital subtraction, an injection of indigo carmine via the catheter was used to determine whether the infused carboplatin was dispersed appropriately through the tumour. The carboplatin was then infused at a dose based either on the patient’s body surface area (350 mg m^−2^), or according to Calvert’s formula (4.5 times the area under the curve, AUC) after renal function had been assessed (Calvert *et al*, 1989; Oya *et al*, 2004). It was administered within about 20 s. When multiple nutrient arteries were present, the dose of carboplatin for each artery was determined based on the percentage of tumour stained with indigo carmine after each vascular infusion. Heparin sodium (2000 U h^−1^) and methylprednisolone (125 mg) were added to the infusions to prevent thromboses and spasms at the catheterisation site. This targeted i.a. chemotherapy was performed after about 10 Gy irradiation. Irradiation was administered by an external beam X-ray targeted to the primary sites. This consisted of doses of 1.5 Gy per fraction, twice daily with a minimum of 6 h between fractions, administered 5 days a week with a total dosage of 30 Gy over 2 weeks.

### Tumour angiogenesis

Tumour specimens in which BVD was to be measured were obtained from diagnostic biopsy specimens before any treatments were begun. Blood microvessels were stained for CD31 protein using a standard immunoperoxidase technique. All sections were processed routinely; sections (4 *μ*m) were cut and counterstained using streptavidin–biotin labelling. Counting was made by a researcher with no knowledge of the treatment for individual patients. Counts were made of the numbers of vessels per × 200 microscope objective field. Angiogenesis was evaluated using an established method (Vermeulen *et al*, 1996). The means and standard deviations of all counts were calculated. Blood vessel density counts were divided into two groups based on the median BVD measured from 117 patients with squamous cell carcinomas of the oral cavity and oropharynx who were treated at our university hospital between June 1986 and February 2002. The counts were recorded as high (⩾11 per field) or low (<11 per field).

### Estimation of response to treatment

To estimate the response to targeted i.a. carboplatin infusion and concurrent radiotherapy, we evaluated formalin-fixed paraffin-embedded sections (4 *μ*m) of tumour tissues taken from all patients at resectional surgery. Sections with no viable tumour cells in the tissue were graded histologically as a complete response (CR); those with at least some viable tumour cells were graded as an incomplete response (ICR).

### Statistics

We used the Mann–Whitney *U*-test and Spearman’s correlation coefficient for nonparametric analysis. We used a logistic regression model to analyse multivariate relationships between BVD and treatment response and other prognostic factors. The survival rates were calculated by the Kaplan–Meier method and compared using log-rank tests. All calculations were performed using the Stat View version 5J software package (Abacus Concepts, Inc., Berkley, CA, USA). Statistical significance was defined as *P*<0.05.

## RESULTS

### Experimental study

**Figure 4:**
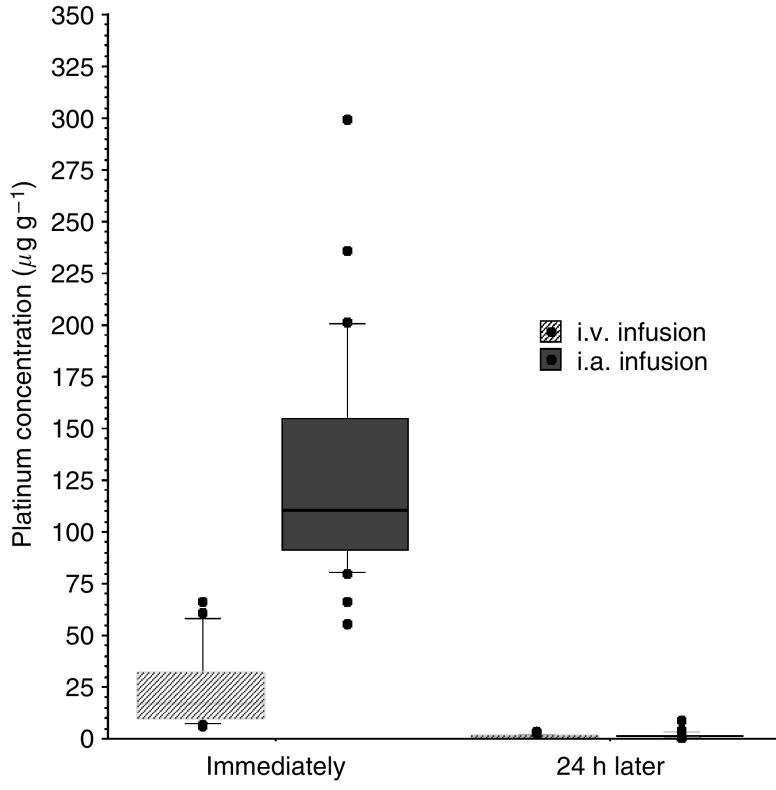
Tissue platinum concentrations of the VX2 tongue tumour region from 46 rabbits after the administration of carboplatin. In comparison with i.v. infusion (*n*=26, median=7.98 *μ*g g^−1^), the i.a. infusion method showed a higher carboplatin concentration in the tongue tissue (*n*=20, median=80.45 *μ*g g^−1^) immediately after the administration of carboplatin. But 24 h after the administration of carboplatin, no difference in the tissue platinum concentration of i.a. and i.v.infusion was observed.

**Figure 5:**
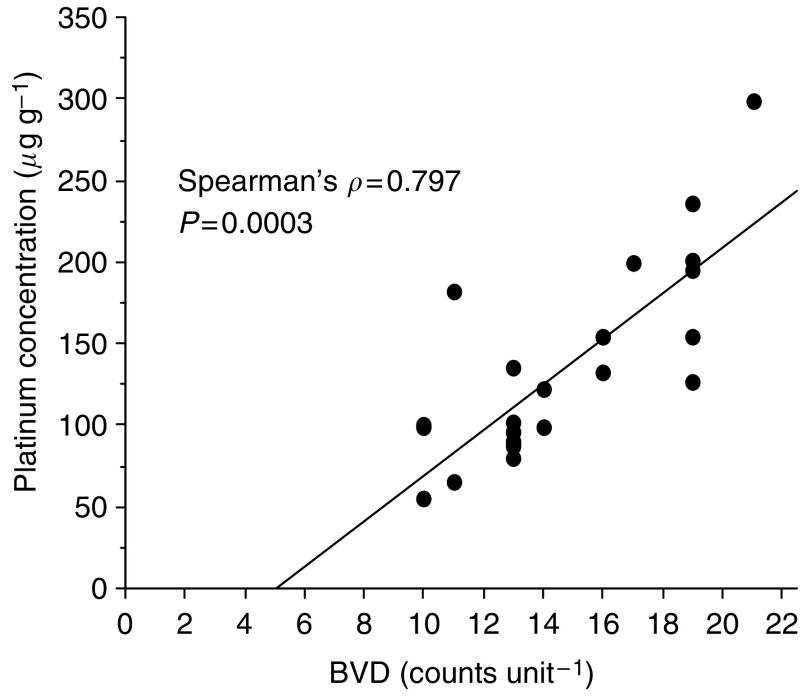
Spearman's correlation coefficients were used to evaluate the increase in the BVD correlated with the free platinum concentration of the tongue tissue. In the i.a. infusion group, the increase in the BVD was associated with a higher free platinum concentration in the tumour region. (Spearman *ρ*=0.797). However, no relationship was observed between the platinum concentration in the i.v. infusion group and in the normal region of the i.a. infusion group with the BVD.

The median BVD in the tumour region of rabbits was 13 counts field^−1^ with a range of 8–21, whereas BVD in the normal region was 7.0 counts field^−1^, with a range of 3–11 (*P*<0.001). The concentrations of platinum in tongue tissue specimens are shown in Figure 4. Levels of the i.a. group were significantly higher than in the i.v. group. The higher BVD was associated with a higher free platinum concentration in the tumour region in the i.a. group (Spearman’s *ρ*=0.797, *P*=0.0003; Figure 5). Platinum concentrations were compared between the VX2 tumours and normal tissue specimens. In both groups, tissue platinum concentrations were higher in the tumours than in the normal regions, and the differences were statistically significant (i.a. group, *P*<0.0001; i.v. group, *P*=0.0059).

**Figure 6:**
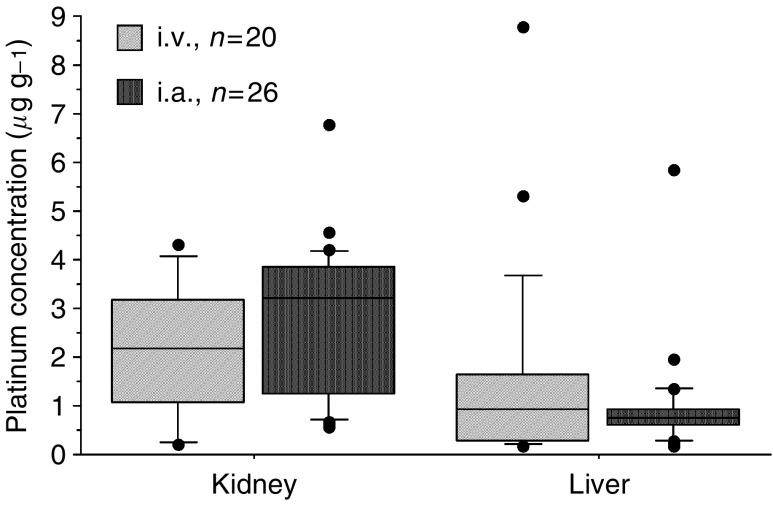
Concentration of platinum in the kidney and the liver at 24 h after the administration of carboplatin. At 24 h, median platinum concentration in the kidney of the i.v. infusion group was 2.19 *μ*g g^−1^, whereas in the i.a. infusion group it was 3.22 *μ*g g^−1^. In the liver, the i.v. infusion group was 0.92 *μ*g g^−1^, whereas the i.a. infusion group was 0.74 *μ*g g^−1^. No statistically significant differences were found regarding the platinum concentrations in the kidney and the liver of the two groups.

To evaluate the carboplatin distribution in other organs according to the infusion routes, we measured platinum concentrations in the kidneys and liver. We found no differences in the platinum concentrations of both organs between the i.a. and i.v. groups (Figure 6).

### Clinical study

#### Distribution of vascularisation counts in biopsy specimens

For the 19 patients reviewed in this study, the median microvessel count for CD31 was 13 counts per × 200 microscope objective field (range, 6–18 counts field^−1^). Twelve patients were classified as a CR and seven as an ICR. The median BVD within the carcinomas of the CR patients was 14 counts field^−1^ (range, 8–18). In contrast, the median BVD in the tumours from ICR patients was 11 counts field^−1^ (range, 6–18 counts/field). The patients were divided into two groups based on BVDs: low-density (⩽11 counts field^−1^) and high-density (>11 counts field^−1^), and the relationship between BVD and the histological response to treatment was examined. In the high-density group, 10 of 12 patients were classed as CR, whereas in the low-density group, two of seven patients were CR (Table 2).

#### Relationship between response to treatment and other prognostic parameters

In the group with tissues staining positive for expressed epidermal growth factor receptor (EGFR), two of seven patients were classed as CR, whereas in the negative EGFR expression group, 10 of 12 patients were CR (*P*=0.0267). No meaningful correlation could be identified with factors such as age, T classification, degree of histological specialisation, pattern of invasion, vascular endothelial growth factor (VEGF) expression, the lowest Hb level during chemoradiation, or histological treatment effects (Table 2).

In a multivariate logistic regression analysis, only BVD was independently related to the treatment effect (*P*=0.0105; odds ratio, 30.00; 95% CI, 2.216–406.083) (Table 3).

**Figure 7:**
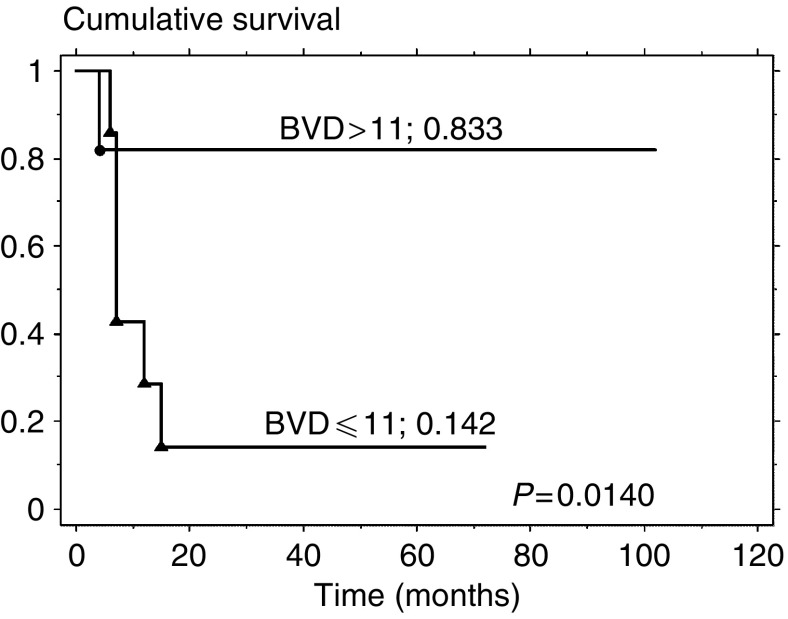
Five-year disease-specific survival curves stratified by BVD status.

**Figure 8:**
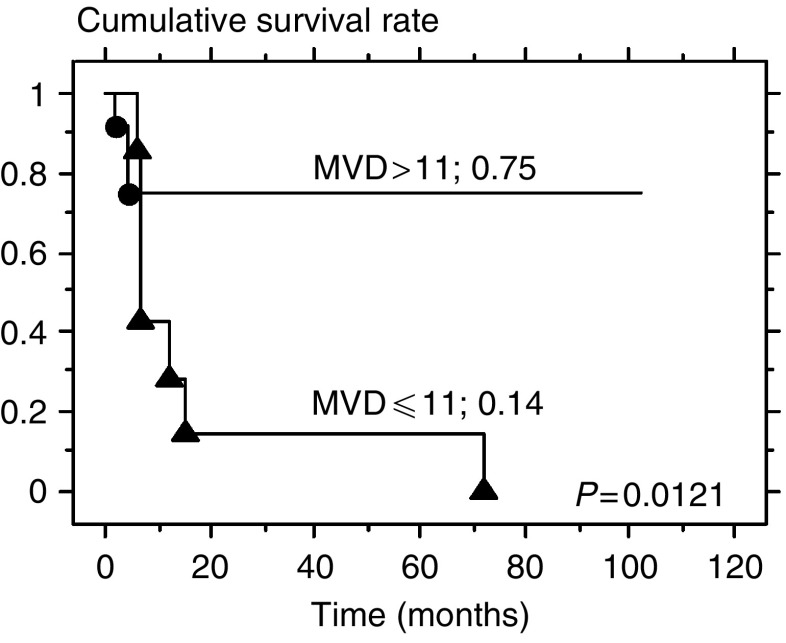
Five-year overall survival curves stratified by BVD.

The 5-year disease-specific survival rate for the patients in the low BVD group was 14%, whereas that for the high BVD group was 83% (*P*=0.014; Figure 7). The 5-year overall survival rate for the patients in the low BVD group was also 14%, but for the high BVD group it was 75% (*P*=0.0121; Figure 8).

## DISCUSSION

In this experimental study, we used a rabbit tongue model of cancer to evaluate targeted i.a. infusion chemotherapy. Many experimental studies using this approach have been reported, but none has used oral tissues and fine-catheter insertion (Figures 1 and 2). We introduced a fine 29-gauge catheter into the lingual artery via the arterial branch of the submandibular gland. This method provided a clear visual field and reliable catheter fixation, allowing us to obtain stable data regarding platinum concentrations in the tongue.

Targeted i.a. injection chemotherapy is considered effective because the anticancer drug is selectively administered via the feeding artery of the tumour and results in a high concentration of drug in the affected tissue. However, many factors affect the distribution of anticancer drugs to tumours. In this study, we paid special attention to the BVD of the tumour as one factor that can influence anticancer drug distribution. Many reports have indicated that BVD is related to both metastasis and survival rate, and BVD has even been considered an index of malignancy in some cancers. However, few reports have addressed the effects of chemotherapy on BVD. Tukuda (1999) reported a relationship between BVD in tumours and the therapeutic effects of neoadjuvant chemotherapy in head and neck cancers. They concluded that the higher the BVD, the easier it is for anticancer drugs to be distributed to the tumour’s focus. Koukourakis *et al* (2000) reported that vascularisation was closely related to the success of chemoradiotherapy, so they designed treatment plans based on the degree of angiogenesis in the tumour. Although the relationship between the distribution of anticancer drugs by i.a. injection and the blood flow of the focus has yet to be clarified, augmentation of the antitumour effects of drugs by increasing local blood flow has already been used clinically.

Among the several factors identified to date, Hb level, EGFR (Neal *et al*, 1990) and VEGF are all regarded as candidate influences on the effects of chemoradiation in the treatment of head and neck cancers. In the present study, EGFR immunostaining in the tumour was found to be a useful factor for making decisions to use targeted chemoradiotherapy. Sainsbury *et al* (1987) reported that, for patients with breast cancer, being EGFR-positive is significantly associated with a poor prognosis. We used immunohistochemistry to visualise EGFR in the tissue. However, this method was difficult to evaluate objectively, and we could not identify a significant relationship between EGFR level and the treatment effect using multivariate analysis.

Strong evidence from retrospective studies suggests that anaemia not only reflects a biologically more aggressive tumour, but that it may be a mediating factor in resistance to radiotherapy (Bush, 1986; Dische, 1991; Obermair *et al*, 2001). In our study, no relationship was observed between Hb levels and the targeted chemoradiation effect. This may have reflected differences between whole body Hb level and that in the tumour, or heterogeneity in oxyhaemoglobin (HbO_2_) saturation within the tissue. Vaupel *et al* (1989) measured HbO_2_ data for erythrocytes from various distributions, and found substantial variations in oxygenation status between four different cryobiopsies of the same metastatic lesion (Vaupel and Kallinowski, 1987). Such heterogeneities might have caused differences in the degree of chemoradiation effect on the tumour cells in the present study.

In this study, we classed two of 12 patients in the high BVD group as having an ICR, and two of the seven patients in the low BVD group showed a CR. Our evidence does not explain this directly, and larger series of patients with oral carcinomas would probably be required to do so. Various factors, which we did not examine in this study, may have affected this result. Some excellent studies on targeted i.a. chemoradiotherapy (Denys *et al*, 1997; Doweck *et al*, 2002; Robbins *et al*, 2004) have reported that primary tumour volume, pathologically positive lymph nodes, tumour regression rate during chemoradiation therapy and total dose of radiation were important factors for predicting treatment outcome among patients with advanced head and neck cancers. In particular, as for lymph node metastases, significant associations with an increase of BVD were reported for various tumours. In the present study, four of the 19 patients died of lymph node metastases, so we intend further studies on this outcome and BVD in the effects of targeted i.a. chemoradiotherapy. One reason was that in this clinical study, we used biopsy specimens selected from the ‘hot spot’ of angiogenesis in each tumour, and this may have biased the estimation of BVD. The other may have been in the relation between the function and size of blood vessels. Hannen and Riediger (2004) reported that large blood vessels in tumours are functional, whereas small ones may represent single endothelial cells without significant perfusion capability. Therefore, there may be regions of tumours with low blood flows and low local O_2_ concentrations despite having high BVD.

## CONCLUSION

Much experimental evidence has shown that tumour growth and metastasis are dependent upon tumour angiogenesis. However, few reports have elucidated the relationship between the effects of chemotherapy and such angiogenesis. Accurately predicting the effects of neoadjuvant chemoradiotherapy for patients with advanced oral cancer is important for the selection of optimal treatment strategies and regimens.

We have identified a close correlation between BVD and the concentration of anticancer drugs in tissues. These data suggest that the BVD of a tumour predicts the effects of targeted carboplatin i.a. chemotherapy for oral cancer.

## Figures and Tables

**Table 1:** Characteristics of the 19 patients studied

Age range (years)	43–78
Mean age (years)	63.1
	
*Sex (number of patients)*	
Male	13
Female	6
	
*Site of primary tumour (number of patients)*	
Tongue	6
Gingiva	7
Buccal mucosa	4
Oral floor	1
Soft palate	1
	
*T stage (number of patients)*	
T2	8
T3	2
T4	9
	
*Haemoglobin level*	
Median. 11.5 g dl^−1^; range: 8.6–15.7	
Low(⩽11.5g dl^−1^)	10
High(>11.5g dl^−1^)	9
	
*Differentiation (number of patients)*	
Poor	10
Moderate	2
Well	7
	
*Pattern of invasion (number of patients)*	
II	3
III	6
IV	10
	
*Expression of EGFR (number of patients)*	
Negative	12
Positive	7
	
*Expression of VEGF (number of patients)*	
Negative	8
Positive	11
	
*MVD*	
Median;13 counts unit^−1^, range; 6–18	
Low (MVD⩽11 counts unit^−1^)	7
High (MVD>11 counts unit^−1^)	12

BVD=blood vessel density; EGFR=epidermal growth factor receptor; VEGF=vascular endothelial growth factor.

**Table 2:** Univariate analysis of clinicopathological factors and response to treatment

**Factor**	**ICR (%)**	**CR (%)**	**Odds ratio**	**95% CI**	***P***
*Age (years)*					
⩽65	4 (41.7)	5 (58.3)			
>65	3 (30.0)	7 (70.0)	1.867	0.283–12.312	0.5166
					
*T stage*					
T1+T2	2(25)	6 (75)			
T3+T4	5 (45.5)	6 (54.5)	0.40	0.055–2.934	0.3674
					
*Haemoglobin level*					
Low (⩽11.5 g dl^−1^)	4 (40.0)	6 (60.0)			
High (>11.5 g dl^−1^)	3 (33.3)	6 (66.7)	1.333	0.311–12.842	0.465
					
*Differentiation*					
Well	3 (42.9)	4 (57.1)			
Moderate+poor	4 (36.4)	8 (63.6)	1.500	0.220–10.220	0.6787
					
*Pattern of invasion*					
1+2+3	3 (33.3)	6 (66.7)			
4	4 (40.0)	6 (60.0)	0.750	0.115–4.899	0.750
					
*Expression of EGFR*					
Negative	2 (16.7)	10(83.3)			
Positive	5 (71.4)	2 (28.6)	0.08	0.009–0.748	0.0267
					
*Expression of VEGF*					
Negative	3 (37.5)	5 (62.5)			
Positive	4 (36.4)	7 (63.6)	0.952	0.144–6.282	0.9596
					
*BVD*					
Low(⩽11)	5 (71.4)	2 (28.6)			
High(>11)	2 (16.7)	10 (83.3)	12.50	1.338–116.82	0.0267

BVD=blood vessel density; CI=confidence interval; CR=complete response; EGFR=epidermal growth factor receptor; ICR=incomplete response; VEGF=vascular endothelial growth factor.

**Table 3:** Multivariate analysis of clinicopathological factors and response to treatment

**Factor**	**Odds ratio**	**95% CI**	***P***
*T stage*			
T1+T2 *vs* T3+T4	0.300	0.030–0.972	0.3036
			
*Haemoglobin level*			
Low *vs* high	1.849	0.180–8.948	0.6047
			
*Expression of EGFR*			
Negative *vs* positive	0.368	0.039–0.474	0.3826
			
*BVD (CD 31)*			
⩽11 *vs* >11	30.00	2.216–406.083	0.0105

BVD=blood vessel density; CI=confidence interval; EGFR=epidermal growth factor receptor; VEGF=vascular endothelial growth factor.
